# Seed priming with ascorbic acid and spermidine regulated auxin biosynthesis to promote root growth of rice under drought stress

**DOI:** 10.3389/fpls.2024.1482930

**Published:** 2024-12-06

**Authors:** Kangkang Zhang, Mohammad Nauman Khan, Zaid Khan, Tao Luo, Biaojin Zhang, Junguo Bi, Liyong Hu, Lijun Luo

**Affiliations:** ^1^ Institute of Quality Safety and Standards of Agricultural Products Research, Jiangxi Academy of Agricultural Sciences, Nanchang, China; ^2^ National Key Laboratory of Crop Genetic Improvement, College of Plant Science and Technology, Huazhong Agricultural University, Wuhan, China; ^3^ Shanghai Agrobiological Gene Center, Shanghai, China; ^4^ College of Natural Resources and Environment, South China Agricultural University, Guangzhou, China

**Keywords:** water-saving and drought-resistance rice (WDR), drought resistance, root morpho-physiological traits, ascorbic acid, spermidine, seed priming, auxin

## Abstract

**Introduction:**

Drought stress severely hampers seedling growth and root architecture, resulting in yield penalties. Seed priming is a promising approach to tolerate drought stress for stand establishment and root development.

**Methods:**

Here, various seed priming treatments, *viz*., hydro priming, ascorbic acid priming (AsA), and spermidine priming (Spd), were adopted concerning root morphological, physiological, microstructural, and molecular studies under drought stress on rice variety Hanyou 73.

**Results and discussion:**

Results demonstrated that drought severely suppressed seedling establishment, while AsA or Spd priming effectively alleviated the inhibitory effects of drought stress, and significantly increased shoot length (24.5-27.9%), root length (34.6-38.8%), shoot dry weight (56.1-97.1%), root dry weight (39.6-40.6%), total root length (47.0-57.8%), surface area (77.0-84.9%), root volume (106.5-109.8%), average diameter (16.4-19.7%), and root tips (46.8-61.1%); meanwhile, priming with AsA or Spd alleviated microscopic and ultrastructural damage from root cell, and improved root activity (183.8-192.0%). The mitigating effects of AsA or Spd priming on drought stress were primarily responsible for decreasing the accumulation of reactive oxygen species by increasing antioxidants activities and osmoprotectants contents, which reduced oxidative stress and osmotic cell potential and facilitated improved water and nutrients absorption in roots. Additionally, seed priming with AsA or Spd substantially improved auxin synthesis by upregulating of *OsYUC7*, *OsYUC11* and, *OsCOW1* expression. However, there were certain differences in the defense responses of plants and mechanisms of reducing the damage of drought stress after seed treatment with AsA or Spd. Under stress conditions, AsA had a greater impact on improving the fresh and dry weight of aboveground parts, while Spd affected the concentration of total sugar and total protein in plants. Likewise, the degree of oxidative damage was lowered, and POD and CAT activities were elevated due to Spd priming under water-deficient conditions.

## 1 Introduction

Rice (*Oryza sativa* L.) is the most important staple food due to its essential sources of food and higher nutritional values for nearly half of the human population worldwide (Bi et al., 2021). As the major producer of rice, China contributes to more than 28% of total global rice production. Therefore, the stabilization of rice production in China plays a crucial role in world food security (Liu et al., 2022). However, drought stress severely affects rice growth, most notably at the germination and early seedling growth stages, which raises the risk of huge yield loss. Therefore, how to improve the drought resistance of rice seedlings is the pivotal issue to be addressed in rice production.

Researchers have taken various ways and means to improve the drought tolerance of crops, including selecting drought-resistant varieties through traditional breeding, cross breeding, genetic engineering, and other methods (Jisha et al., 2013), such as adopting various water-saving irrigation and cultivation techniques and applying different exogenous substances to improve plant drought resistance. Seed priming is an efficient and practical approach to promote seed germination, stand establishment, and stress tolerance in many field crops, particularly under undesirable growth conditions (Zhang et al., 2022a, 2022). Seed priming, a type of pre-sowing seed treatment, controls the seed hydration to a point where the pre-germination metabolisms are activated but prevents radical protrusion from the seed coat (Zhang et al., 2022a). Various chemical molecules, including biostimulants (Jisha et al., 2013; Shukla et al., 2015), organic acids (Wang et al., 2019; Zhang et al., 2022a), and phytohormones (Khan et al., 2019; Zhang et al., 2022b), are commonly applied in seed priming. Applications of biopriming with *Trichoderma* effectively alleviated drought damage to wheat (Shukla et al., 2015), whereas seed priming with wood vinegar greatly mitigated the adverse impacts of drought on upland rice (Zhang et al., 2022a). Moreover, melatonin (Khan et al., 2019), gibberellic acid (Zhang et al., 2022b), and salicylic acid (Fan et al., 2022) were also demonstrated to be effective against drought-induced stress.

There has been increasing evidence that primed plants display increased root weight, better root morphology, and decreased ROS contents due to the activation of cellular defense responses, which impart resistance to exposure to environmental stresses (Ur Rahman et al., 2021). Recently, ascorbic acid (AsA) or spermidine (Spd) as the seed priming agent has been confirmed to enhance crop resistance to abiotic stress. AsA is one of the essential metabolites involved in cell division and osmotic adjustment and possesses a strong antioxidant capacity, facilitating the balance of ROS production and scavenging (Farooq et al., 2013). For example, AsA priming was reported to effectively lower the accumulation of H_2_O_2_ and malondialdehyde (MDA) and consequently enhance seedling growth and drought tolerance (Salemi et al., 2019). As one of the major components of polyamine, spermidine (Spd) can protect macromolecules and maintain the metabolic levels of polyamine by eliminating oxygen free radicals, thereby elevating the osmotic potential, ion homeostasis, and antioxidant system of plant cells under abiotic stress (Hongna et al., 2021). Previous research suggested that seed priming with Spd successfully alleviated various abiotic stresses and prevented the cell structure of the plants from drought (Li et al., 2014), salinity (Hongna et al., 2021), and chilling (Sheteiwy et al., 2017).

In recent years, more attention has been paid to the research of plant roots, because not only roots are the initial receptors that send out water shortage signals (Wang et al., 2019) but also their shape and size are closely related to the drought resistance of plants. It has been widely reported that plants form a huge root system via enhancing the amount of roots, thereby improving the uptake capacity of water and minerals and increasing drought resistance (Battisti and Sentelhas, 2017). Root development is affected by varying endogenous and exogenous factors, like indoleacetic acid (IAA), which is the most well-known hormone that demonstrates a vital role in regulating almost every aspect of root growth and development (Cao et al., 2019). Auxin is primarily synthesized by the tryptophan aminotransferase of arabidopsis (TAA)/YUCCA (YUC) pathway, which is highly conserved in the plant kingdom (Zhao, 2012). In rice, 2 and 14 gene members belong to the TAA and YUC families, respectively (Woo et al., 2007; Yoshikawa et al., 2014), which involves major biological processes mediated by the activity of auxin. Previously, it has been documented that the biosynthesis of IAA mediated by YUC is critical for adaptation to drought stress in numerous plants, for example, Arabidopsis (Cha et al., 2015), potato (Kim et al., 2013), and rice (Woo et al., 2007).

Water-saving and drought-resistance rice (WDR) is an innovative rice cultivar that is characterized by higher yield, excellent grain quality, and less water consumption (Luo, 2010). In recent years, 22 WDR varieties were released and distributed to farmers. They could be grown in both irrigated and rainfed ecosystems in China, where the total planted area has accumulated to more than one million hectares (Luo et al., 2019). Furthermore, it has been reported that the better growth and yield performance of WDR may be attributed to more robust root systems under water shortage conditions (Bi et al., 2021). However, there is little literature available to explore the responses of root morphology, ultrastructure, physiology, and molecular induced by seed priming and to unravel the mechanisms of priming-induced stress tolerance in WDR. Therefore, the objectives of the current study were to investigate the impacts of seed priming on root morphology, microstructure, and physiology of WDR plants under drought stress and to reveal the underlying mechanisms of priming-induced root morphological changes under drought stress in terms of IAA contents and relative gene transcription.

## 2 Materials and methods

### 2.1 Experimental materials

Seeds of widely grown WDR cultivar, Hanyou 73 (HY73, hybrid), obtained from Shanghai Agrobiological Gene Center (SAGC, Shanghai, China) with a germination rate of ≥ 90% were used in this study. AsA and Spd, the seed priming agent, were purchased from Shanghai Yuanye Biotechnology Co., Ltd., Shanghai, China. The effective concentration of AsA (100 mg/L) and Spd (4 mM) was pre-optimized on the basis of the performance of seed germination and seedling growth.

### 2.2 Experimental design

Several treatments designated for the current study included: [1] no priming + no stress (NP + NS), [2] no priming + drought stress (NP + DS), [3] hydro priming + drought stress (HP + DS), [4] AsA priming + drought stress (AsA + DS), and [5] Spd priming + drought stress (Spd + DS). Clean, healthy seeds were surface-sterilized using 1% (v/v) NaClO solution for 15 min and then rinsed three times with distilled water. The sterilized seeds dried with blotted paper were primed for 24 h in the dark at 25°C in distilled water (hydro priming) or AsA (100 mg/L) or Spd (4 mM) solution. The seed weight ratio to the priming solution volume (w/v) was 1:5. After 24 h, primed seeds were cleaned with distilled water three times, blotted dried, and kept at 25°C until the original weight was achieved.

The experiments were carried out in the growth chamber (HP250GS-C, Ningbo Southeast Instrument Co., Ltd., Ningbo, China) with 12-h light (8,000 lx) and day/night temperature at 30°C/25°C. Forty seeds were sowed in plastic pots (24 × 17 × 8.5 cm) containing 1 L of distilled water for no stress (NS) or 15% PEG-6000 solution (w/v) for drought stress (DS) treatments. The floating board on the surface of the solution contained four separate sections. All the treatments were conducted in a completely randomized design with six replications and were repeated thrice to determine physiological and biochemical traits. Seedlings were evaluated at 9 days after sowing.

### 2.3 Shoot length, root length, and seedling weight

The shoot length and root length of 10 randomly sampled seedlings were measured in each treatment. The selected seedlings were separated into shoots and roots to record their fresh weight (FW) and then were oven-dried at 75°C for 48 h to weigh shoot dry weight (DW) and root DW, respectively.

### 2.4 Seedling root morphology

The roots of five fresh seedlings from each treatment were scanned with an Epson V800 scanner (Epson Seiko Epson Corporation, Nagano Prefecture, Suwa, Japan) at 300 dpi and then oven-dried to obtain root DW. Root morphological parameters, such as total root length, surface area, root volume, average diameter, and root tips, were then assessed by WinRHIZO 2017a software (Regent Instruments, Quebec City, QC, Canada) (Zhang et al., 2022a). Specific root length was calculated by dividing the total root length by root DW, namely, specific root length (cm mg^−1^ DW) = total root length/root DW per seedling.

### 2.5 Anatomical structure of roots

Fresh root tips excised from the rice seedlings were fixed with Formaldehyde-acetic acid-ethanol (FAA) solution. These segments were embedded in agarose solution and frozen immediately and sectioned at 16 μm. Subsequently, the slices were stained with 0.1% aniline blue dye and examined using an Eclipse E100 microscope (Nikon, Japan).

### 2.6 Root ultrastructure

Fresh root tips from each treatment were sampled and fixed in 4% glutaraldehyde and 0.2 M sodium phosphate buffer (pH 6.8), followed by washing with the same buffer three times. Samples were post-immobilized in 1% osmic acid in 0.2 M sodium phosphate buffer (pH 6.8), dehydrated in a graded ethanol series, and dried with critical point drying. Extremely thin sections were stained with lead citrate and 2% uranyl acetate and eventually were viewed by a transmission electron microscope (Hitachi H-7650).

### 2.7 Root activity, soluble sugar, soluble protein, and free proline

Fresh root samples (about 0.2 g) were collected, root activity was evaluated by the triphenyl tetrazole chloride method, and the absorbance was recorded at 485 nm (Zhang et al., 2013). The osmoprotectants, such as total soluble sugar, soluble protein, and free proline contents in the root tissues, were measured by using the kits of Nanjing Jiancheng Bioengineering Institute, Nanjing, China. Briefly, 0.2 g of root samples were milled with a motor and pestle and mixed with 1.8 mL of distilled water under chilled conditions. The mixture was centrifuged at 12,000 rpm for 10 min at a low temperature. The supernatant was collected to determine soluble sugar, soluble protein, and free proline contents according to the introduction of the kits.

### 2.8 H_2_O_2_, ${\text{O}}_{2}^{.-}$ , malondialdehyde, and electrolyte leakage

H_2_O_2_, ${\text{O}}_{2}^{.-}$ and MDA of root samples were performed according to kits provided by Nanjing Jiancheng Bioengineering Institute, Nanjing, China. Briefly, 0.2 g of fresh roots were ground to powder in distilled water. The supernatants after centrifugation were used for the content of H_2_O_2_, ${\text{O}}_{2}^{.-}$ , and MDA with the help of the kits. Electrolyte leakage (EL) was estimated according to the formula below:


EL=EC1/EC2×100

where EC1 is the initial conductivity and EC2 is the final conductivity (Dionisio-Sese and Tobita, 1998).

### 2.9 Antioxidant enzyme activity

To measure the enzyme activity of antioxidants, i.e., superoxide dismutase (SOD), catalase (CAT), peroxidase (POD) and ascorbate peroxidase (APX), 0.2 g of fresh roots were homogenized with 1.8 mL of 50 mM phosphate buffer (pH 7.8). The mixture was centrifuged to extract the supernatants. The activity of SOD, POD, CAT, and APX was determined by using “SOD Detection Kit,” “POD Detection Kit,” “CAT Detection Kit,” and “APX Detection Kit,” respectively, purchased from Nanjing Jiancheng Bioengineering Institute, Nanjing, China.

### 2.10 Endogenous IAA content

Endogenous IAA levels in the roots of rice plants were performed using the enzyme-linked immunosorbent assay (ELISA) kit provided by the MEIBIAO BIOLOGY company. Briefly, about 0.05 g of root samples were ground with phosphate buffer and centrifuged at 12,000 rpm at 4°C to extract the fine supernatant. The contents of IAA were then quantified with the standard procedure according to the manufacturer. Finally, the absorbance at 450 nm was recorded with the help of the microplate reader.

### 2.11 Total RNA isolation and quantitative real-time PCR

Total RNA was isolated from roots of WDR plants using TRIzol reagent (Servicebio, Wuhan, China). Total RNA of 2 μg was then reverse-transcribed into cDNA with a RevertAid reverse transcription kit (Servicebio, Wuhan, China). The quantitative real-time PCRs of each gene were performed on a SYBR Green monitored quantitative PCR (Servicebio, Wuhan, China). Relative expression levels were measured by the comparative Ct method (Livak and Schmittgen, 2001). The rice Actin gene was used as an internal control. The primers used in the present study can be found in **Supplementary Table S1**.

### 2.12 Statistical analysis

All the data were analyzed using Statistix 9.0 from three biological replicates. The mean-variance of the treatment was compared using the least significant difference (LSD) test at the 0.05 probability level. Graphical presentation of the data was performed with the ggplot2 package in RStudio.

## 3 Results

### 3.1 Effects of AsA and Spd priming on root length, shoot length, and seedling weight

**Figure 1:**
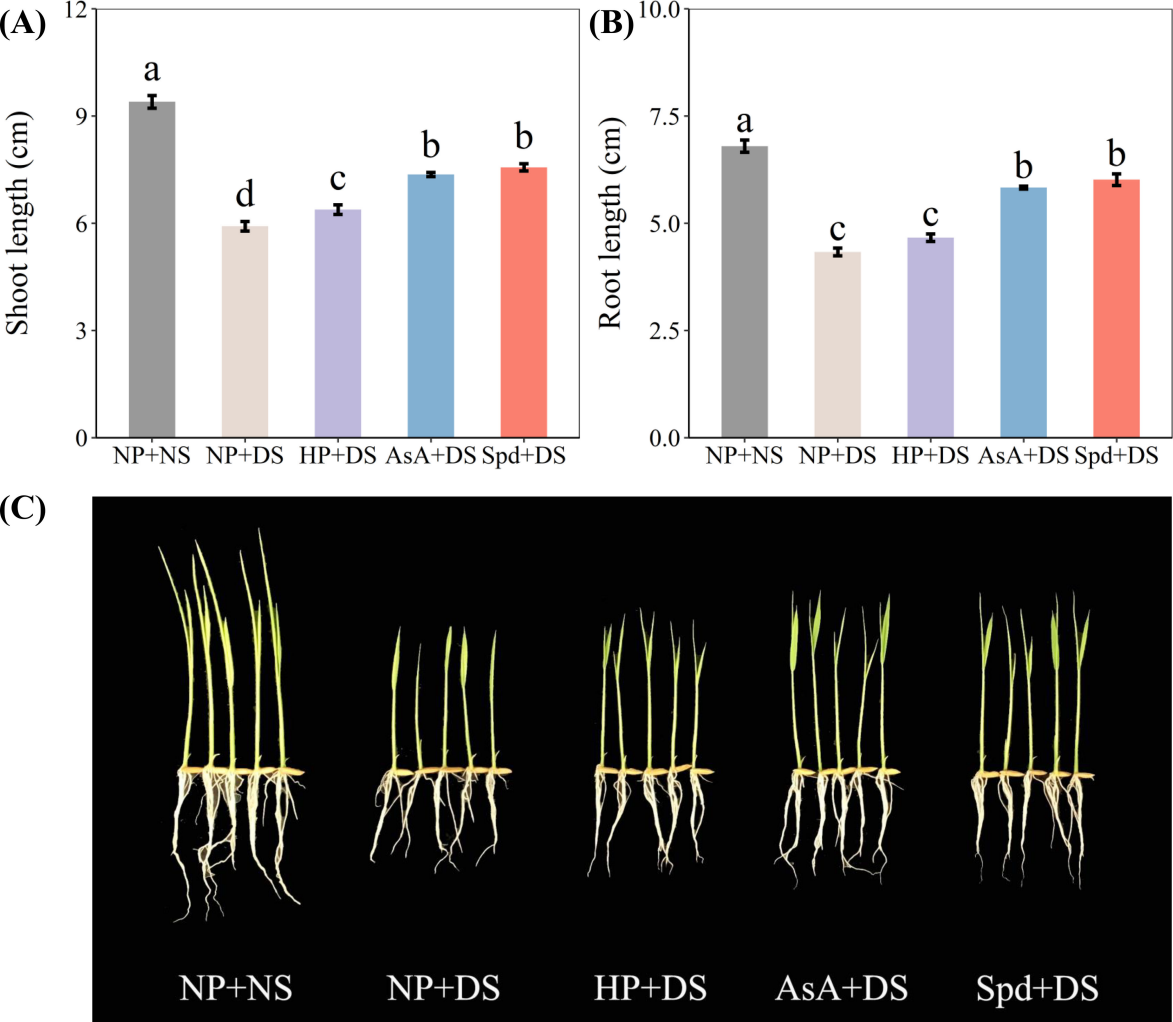
Effect of seed priming on **(A)** shoot length, **(B)** root length, and **(C)** seedling photographs of WDR under drought stress. Different letters show a significant difference between treatments at *P* < 0.05 according to the LSD test. Error bars represent the standard error of three replicates. NP + NS, no priming and no stress; NP + DS, no priming and drought stress; HP + DS, hydro priming and drought stress; AsA + DS, AsA priming and drought stress; Spd + DS, Spd priming and drought stress.

Drought stress significantly increased the seedling attributes, whereas all priming treatments effectively improved the shoot length and root length, except for HP + DS in root length (**Figure 1**). Compared with NP + NS, NP + DS decreased the shoot length and root length by 37.1% and 36.3%, respectively. AsA + DS and Spd + DS enhanced the shoot length by 24.5% and 27.9%, respectively, when compared with NP + DS, whereas the respective increments in root length were 34.6% and 38.8%, respectively. Although HP + DS was the least effective treatment, it improved markedly shoot length by 7.9% compared to NP + DS.

**Figure 2:**
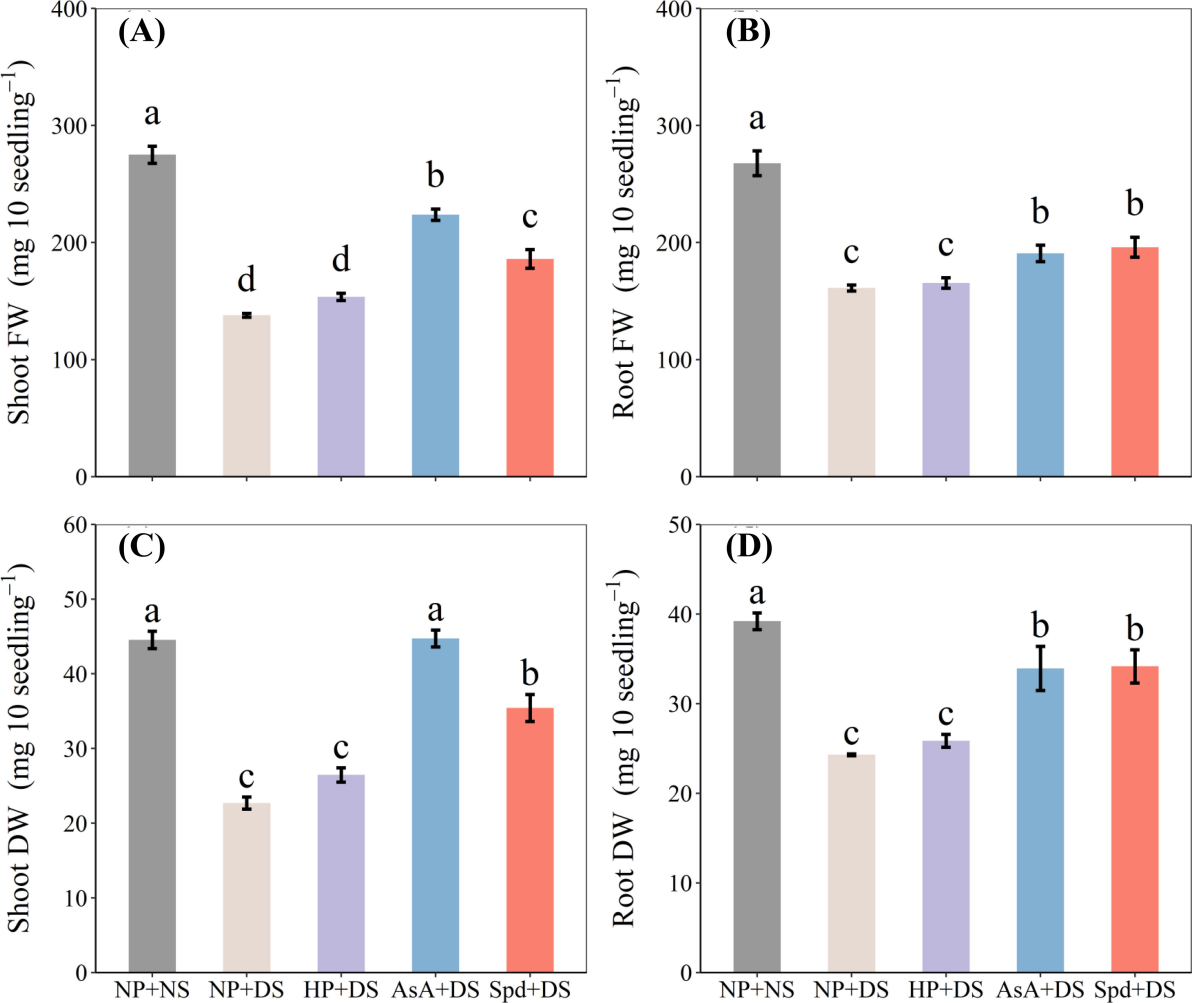
Effect of seed priming on **(A)** shoot FW, **(B)** root FW, **(C)** shoot DW, and **(D)** root DW of WDR under drought stress. Different letters show a significant difference between treatments at *P* < 0.05 according to the LSD test. Error bars represent the standard error of three replicates. NP + NS, no priming and no stress; NP + DS, no priming and drought stress; HP + DS, hydro priming and drought stress; AsA + DS, AsA priming and drought stress; Spd + DS, Spd priming and drought stress; FW, fresh weight; DW, dry weight.

The exposure of seedlings to drought stress resulted in considerably reduced weight, whereas both AsA + DS and Spd + DS were effective in alleviating the drought-induced inhibition of growth (**Figure 2**). Compared with those in NP + NS, shoot FW, root FW, shoot DW, and root DW in NP + DS were decreased by 49.9%, 39.8%, 49.0%, and 38.0%, respectively. Under drought conditions, AsA + DS treatment improved shoot FW, root FW, shoot DW, and root DW by 62.3%, 18.4%, 97.1%, 39.6%, respectively, compared to NP + DS treatment, whereas the corresponding increases in Spd + DS treatment were 34.9%, 21.6%, 56.1%, and 40.6%, respectively. Likewise, AsA + DS recorded significantly higher biomass compared to Spd + DS (**Figures 2A, C**).

### 3.2 Effects of AsA and Spd priming on root morphological traits

**Figure 3:**
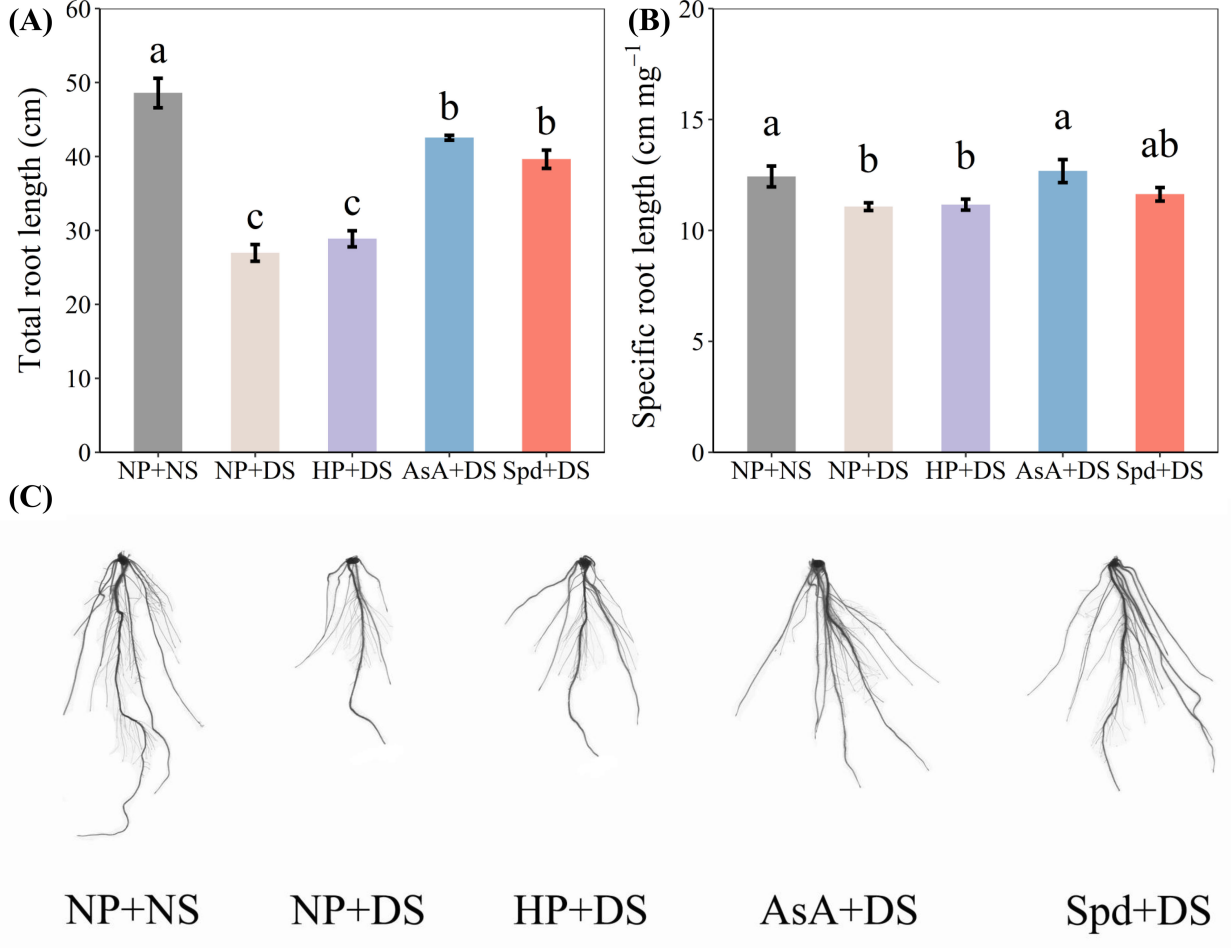
Effect of seed priming on **(A)** total root length, **(B)** specific root length, and **(C)** root photographs of WDR under drought stress. Different letters show a significant difference between treatments at *P* < 0.05 according to the LSD test. Error bars represent the standard error of three replicates. NP + NS, no priming and no stress; NP + DS, no priming and drought stress; HP + DS, hydro priming and drought stress; AsA + DS, AsA priming and drought stress; Spd + DS, Spd priming and drought stress.

**Figure 4:**
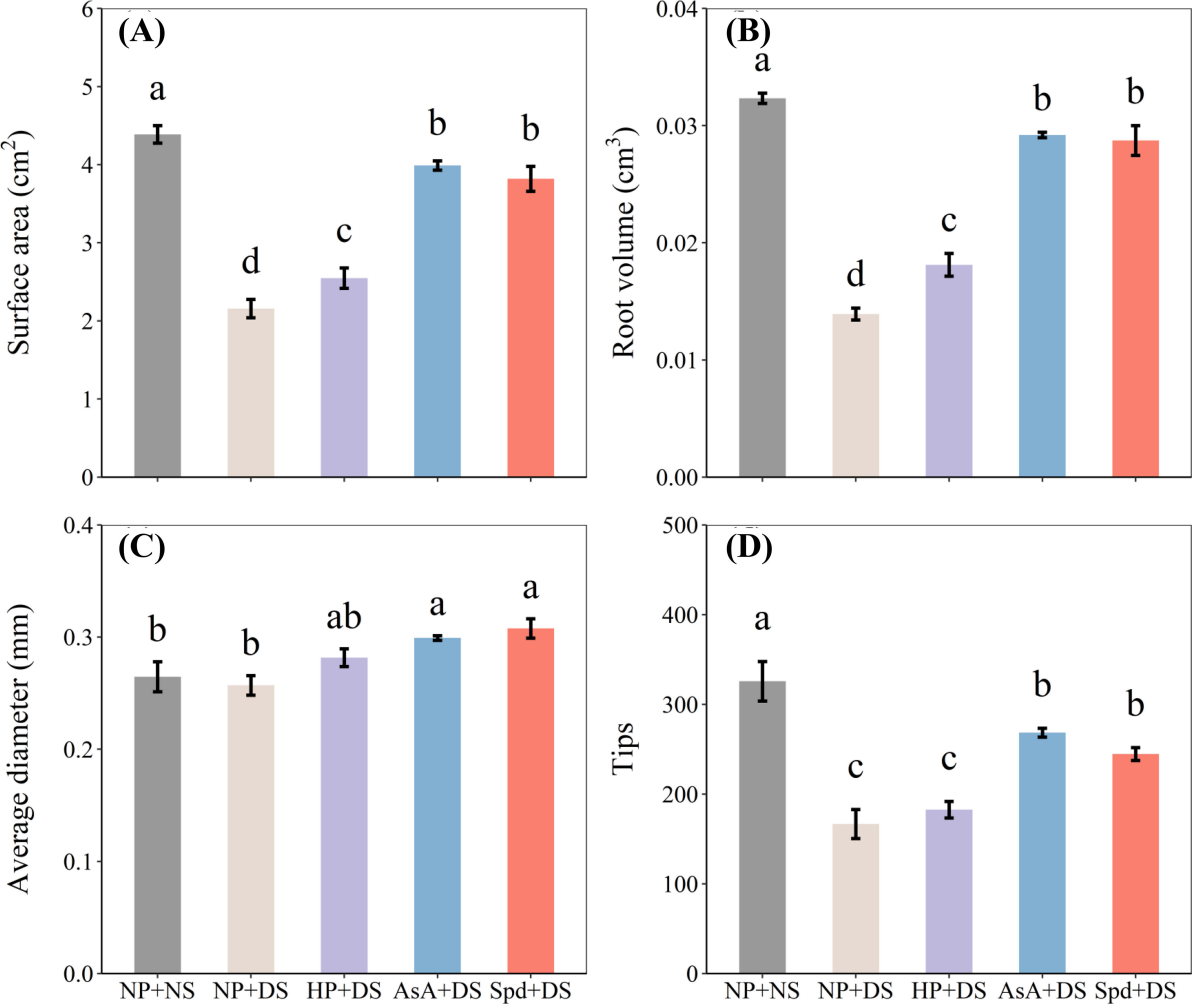
Effect of seed priming on **(A)** surface area, **(B)** root volume, **(C)** average diameter, and **(D)** tips of WDR in roots under drought stress. Different letters show a significant difference between treatments at *P* < 0.05 according to the LSD test. Error bars represent the standard error of three replicates. NP + NS, no priming and no stress; NP + DS, no priming and drought stress; HP + DS, hydro priming and drought stress; AsA + DS, AsA priming and drought stress; Spd + DS, Spd priming and drought stress.

Drought stress was observed to severely hamper root development, whereas AsA and Spd priming treatments improved the parameters of root morphology (**Figures 3**, **4**). When compared with NP + NS, NP + DS lowered total length and specific root length by 44.5% and 11.0%, respectively (**Figures 3A, B**). Under drought stress, the total length in AsA priming and Spd priming was markedly enhanced by 57.8% and 47.0%, respectively. In contrast, significantly higher specific root length was only recorded in the AsA priming treatment, which was improved by 14.5% compared with no priming.

Compared with no stress, no priming treatment under drought stress reduced surface area, root volume, and tips by 50.8%, 57.0%, and 48.8%, respectively (**Figure 4**). Compared to no priming under stress conditions, AsA priming and Spd priming treatment effectively increased surface area by 84.9% and 77.0%, root volume by 109.8% and 106.5%, average diameter by 16.4% and 19.7%, and tips by 61.1% and 46.8%, and these two treatments were statistically similar with each other. Hydro priming partially improved root morphological traits and exhibited an obvious enhancement in surface area by 18.1% and root volume by 30.2% compared with no priming treatment (**Figures 4A, B**). Additionally, compared with AsA priming, Spd priming slightly promoted total root length, specific root length, surface area, and root tips under water-stressed conditions.

### 3.3 Effects of AsA and Spd priming on root microstructure

**Figure 5:**
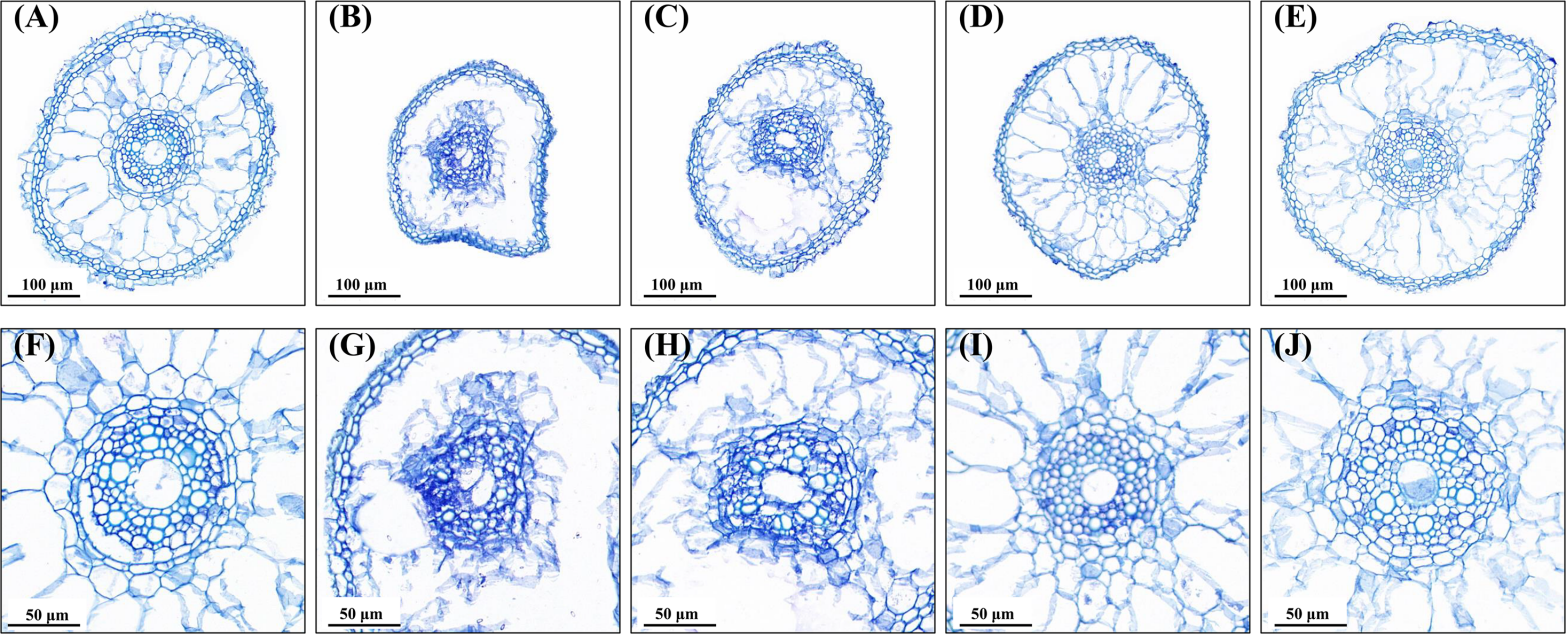
Effect of seed priming on root microstructure of WDR under drought stress. **(A, F)** NP + NS, no priming and no stress; **(B, G)** NP + DS, no priming and drought stress; **(C, H)** HP + DS, hydro priming and drought stress; **(D, I)** AsA + DS, AsA priming and drought stress; and **(E, J)** Spd + DS, Spd priming and drought stress. **(A–E)** ×60 magnification; scale bars, 100 μm; **(F–J)** ×120 magnification; scale bars, 50 μm.

Compared to no water stress (**Figure 5A**), drought stress resulted in severe deformation and shrinkage (**Figure 5B**). More epidermis cells lost water and became smaller in size, and cortical cells were seriously disrupted; additionally, a vague and deformed vascular system was detected under drought stress (**Figure 5G**). However, the anatomical structure of root tips was improved by all priming treatments when exposed to water stress (**Figures 5C–E, H–J**). In comparison with no priming treatment, root sections with AsA and Spd priming remained relatively intact (**Figures 5E, F**). The better root microstructure in the epidermis, cortex, and vascular structure was observed under priming with AsA and Spd (**Figures 5D, E, I, J**), which indicated priming agents maintain the normal framework of root cells. Moreover, seedling roots primed with Spd displayed larger size and clearer cell structure compared to roots treated with AsA.

### 3.4 Effects of AsA and Spd priming on root ultrastructure

**Figure 6:**
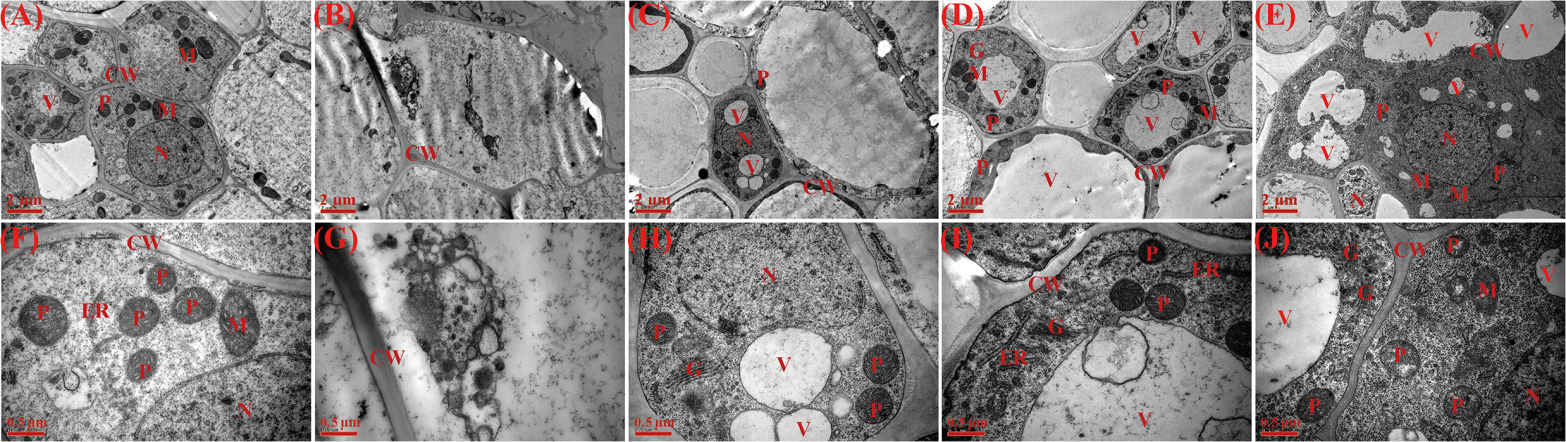
Effect of seed priming on root ultrastructure of WDR under drought stress. **(A, F)** NP + NS, no priming and no stress; **(B, G)** NP + DS, no priming and drought stress; **(C, H)** HP + DS, hydro priming and drought stress; **(D, I)** AsA + DS, AsA priming and drought stress; and **(E, J)** Spd + DS, Spd priming and drought stress. **(A–E)** Scale bars, 2 μm; **(F–I)** scale bars, 0.5 μm. CW, cell wall; V, vacuole; M, mitochondria; N, nucleus. ER, endoplasmic reticulum; P, plastid; G, golgi apparatus.

Transmission electron microscopy (TEM) analysis revealed that, compared with normal water (**Figures 6A, F**), root tip cells under drought stress exhibited distorted morphology, unclear nucleoli, and disordered cytoplasm. Furthermore, under drought conditions, untreated control cells showed almost no observable organelles (**Figures 6B, G**). In contrast, all priming treatments significantly improved the ultrastructure of root tips under water stress (**Figures 6C–E, H–J**). The TEM micrographs of root tips primed with AsA and Spd showed that clear and rich organelle, large, and abundant vacuoles can be observed (**Figures 6D, E**). Furthermore, the cytoplasm in root cells of AsA priming contained endoplasmic reticulum, golgi apparatus, and rich plastid (**Figure 6I**), whereas Spd priming had a large size and well-developed nucleus, more mitochondria, golgi apparatus, and plastid (**Figures 6E, J**). Generally, the cells of root tips with Spd priming showed larger nucleus and abundant organelles compared to those with AsA priming.

### 3.5 Effects of AsA and Spd priming on root activity, soluble sugar, soluble protein and proline

**Figure 7:**
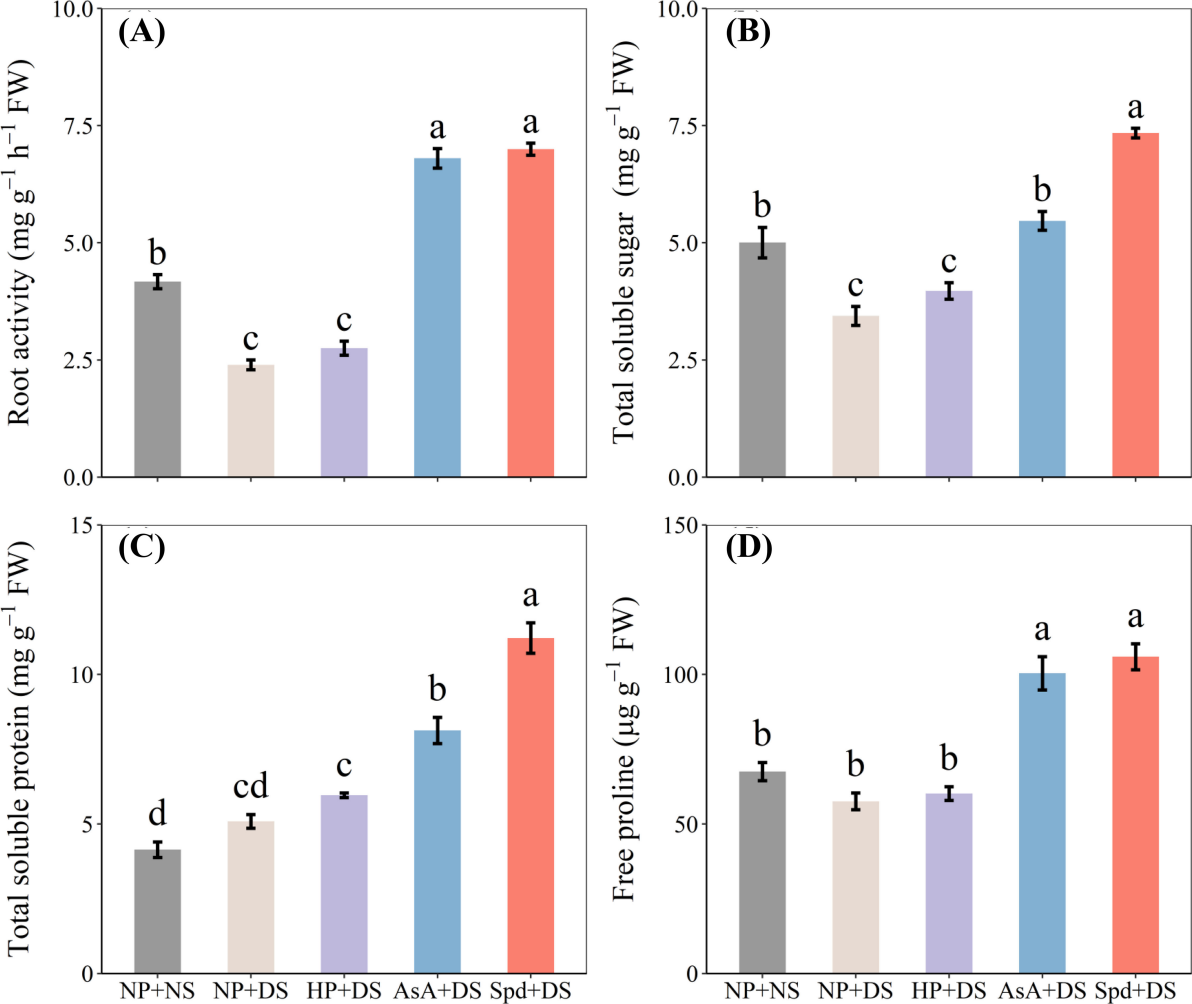
Effect of seed priming on **(A)** root activity, **(B)** total soluble sugar, **(C)** total soluble protein, and **(D)** free proline of WDR in roots under drought stress. Different letters show a significant difference between treatments at *P* < 0.05 according to the LSD test. Error bars represent the standard error of three replicates. NP + NS, no priming and no stress; NP + DS, no priming and drought stress; HP + DS, hydro priming and drought stress; AsA + DS, AsA priming and drought stress; Spd + DS, Spd priming and drought stress.

When compared with NP + NS, NP + DS significantly lowered root activity and total soluble sugar by 42.5% and 31.2%, respectively (**Figures 7A, B**). With exposure to drought stress, AsA priming and Spd priming treatment showed the highest root activity and soluble sugar content, which was higher than no priming treatment by 183.8% and 192.0% and by 58.9% and 113.3%, respectively. There was no significant variance between drought stress and no stress regarding total soluble protein and free proline content (**Figures 7C, D**). When compared with NP + DS, the accumulation of total soluble protein and free proline in AsA + DS and Spd + DS was pronouncedly promoted by 59.7% and 120.4% and by 74.3% and 83.9%, respectively. Additionally, Spd + DS was the most effective treatment for the increase of sugar and protein.

### 3.6 Effects of AsA and Spd priming on H_2_O_2_, ${\text{O}}_{2}^{.-}$ , MDA and electrolyte leakage

**Figure 8:**
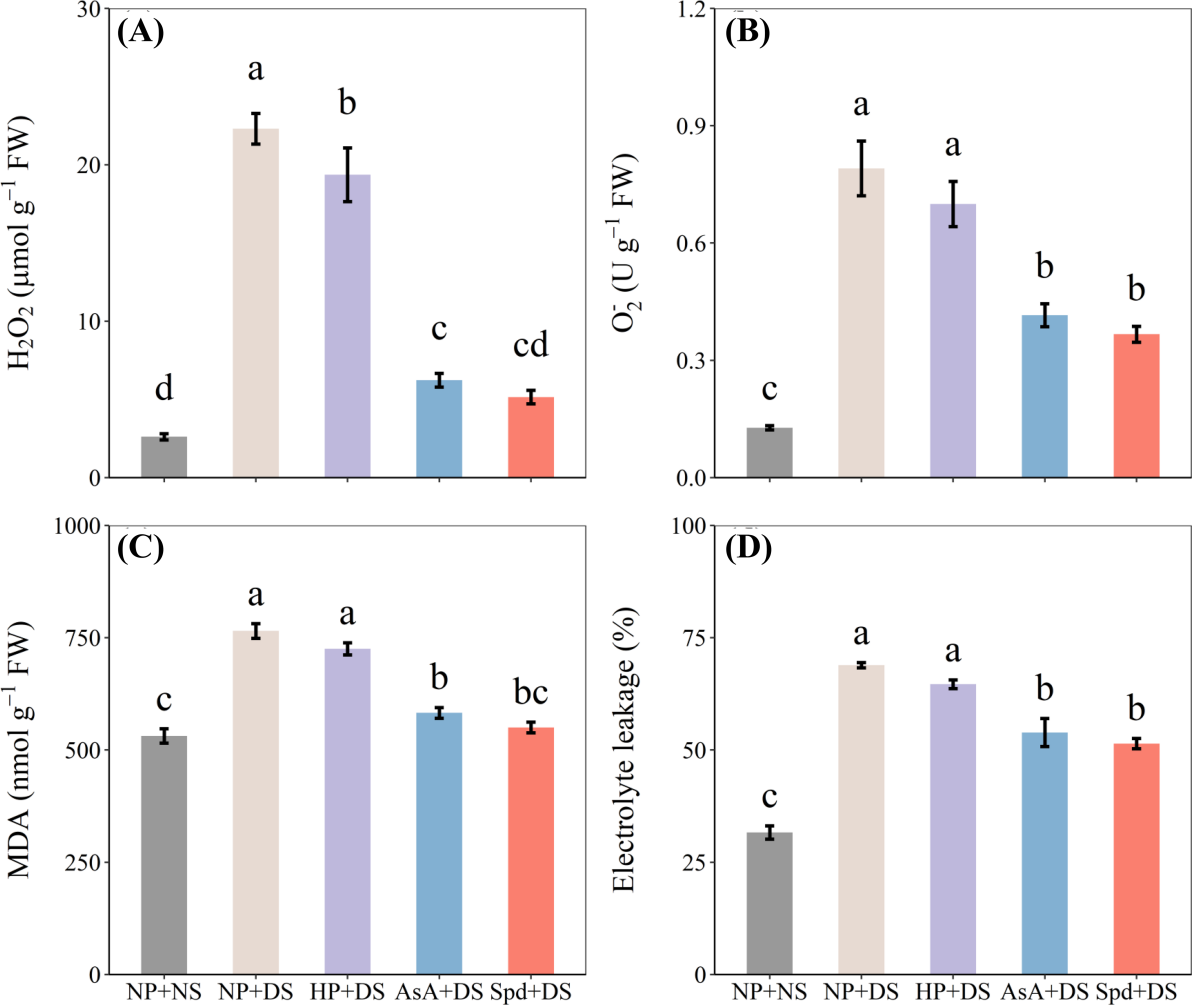
Effect of seed priming on **(A)** H_2_O_2_, **(B)** ${\text{O}}_{2}^{.-}$ , **(C)** MDA, and **(D)** electrolyte leakage of WDR in roots under drought stress. Different letters show a significant difference between treatments at *P* < 0.05 according to the LSD test. Error bars represent the standard error of three replicates. NP + NS, no priming and no stress; NP + DS, no priming and drought stress; HP + DS, hydro priming and drought stress; AsA + DS, AsA priming and drought stress; Spd + DS, Spd priming and drought stress.

Drought stress demonstrated a remarkable increase in H_2_O_2_ and ${\text{O}}_{2}^{.-}$ content compared with NP+NS (**Figures 8A, B**). However, different priming treatments showed favorable effects on minimizing oxidative stress under water stress. The H_2_O_2_ and ${\text{O}}_{2}^{.-}$ contents were decreased by 72.1% and 47.5% in AsA priming treatment, 76.9% and 53.6% in Spd priming treatment, compared to no priming treatment. The exposure to drought stress caused a notable increase in MDA content by 44.0% and EL by 117.7% in roots over no stress treatment (**Figures 8C, D**). AsA priming and Spd priming treatment exhibited a greatly lower MDA and EL under water stress conditions, which declined the MDA concentration by 23.8% and 28.1% and EL by 21.8% and 25.3% as compared with NP+DS treatment. Spd treatment under drought stress reduced the oxidative stress of seedlings compared to AsA treatment, although these two treatments were statistically similar.

### 3.7 Effects of AsA and Spd priming on antioxidant enzymes activities

**Figure 9:**
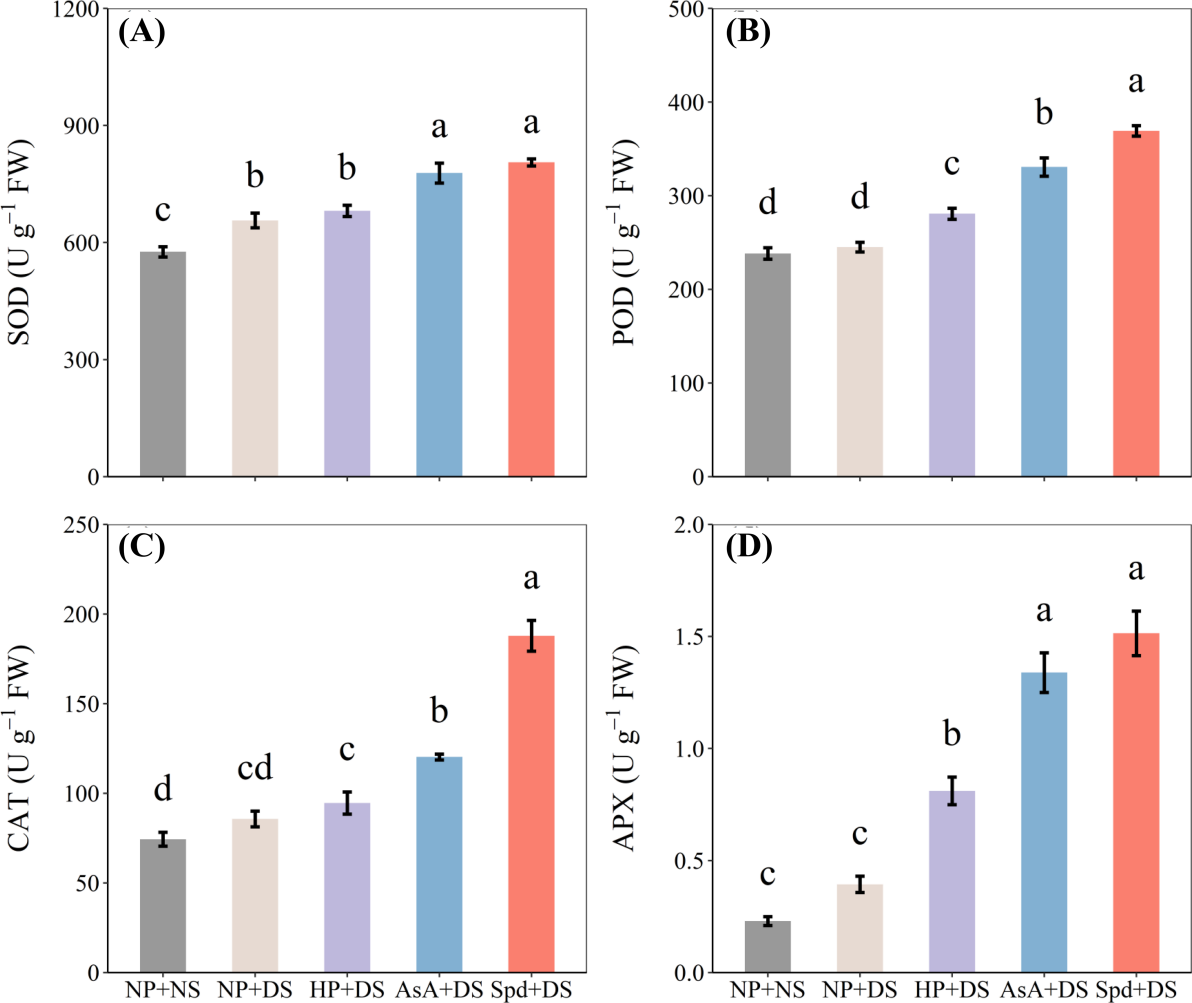
Effect of seed priming on **(A)** SOD, **(B)** POD, **(C)** CAT, and **(D)** APX of WDR in roots under drought stress. Different letters show a significant difference between treatments at *P* < 0.05 according to the LSD test. Error bars represent the standard error of three replicates. NP + NS, no priming and no stress; NP + DS, no priming and drought stress; HP + DS, hydro priming and drought stress; AsA + DS, AsA priming and drought stress; Spd + DS, Spd priming and drought stress.

Compared with NP + NS, the activities of antioxidant enzymes were unaffected by drought stress (NP + DS), except for SOD activity, which was significantly promoted by 13.9% (**Figure 9A**). Under water stress conditions, all priming treatments greatly elevated the activities of antioxidant enzymes, except for SOD and CAT activities in HP + DS treatment (**Figures 9A–D**). AsA priming treatment increased SOD, POD, CAT, and APX activities by 18.5%, 34.9%, 40.3%, and 239.9%, respectively, compared to NP + DS treatment, whereas the respective increases in Spd priming treatment were 22.7%, 50.6%, 56.1%, and 284.5%, respectively. Likewise, Spd + DS was more effective in promoting these antioxidants than all other seed priming treatments.

### 3.8 Effects of AsA and Spd priming on IAA content and relative expression of IAA genes

**Figure 10:**
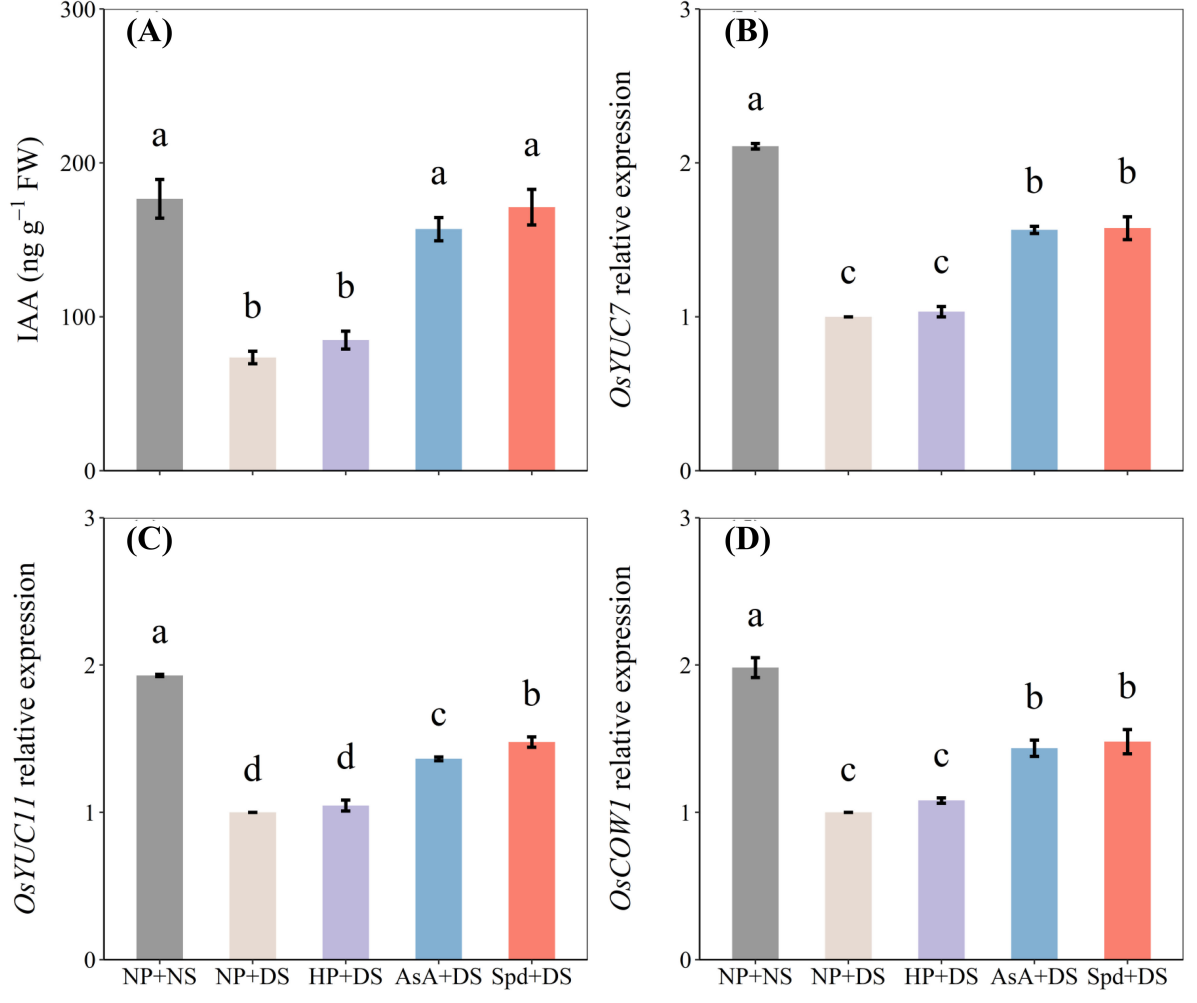
Effect of seed priming on **(A)** IAA content, and the relative expression levels of **(B)** *OsYUC7*, **(C)** *OsYUC11*, and **(D)** *OsCOW1* of WDR in roots under drought stress. Different letters show a significant difference between treatments at *P* < 0.05 according to the LSD test. Error bars represent the standard error of three replicates. NP + NS, no priming and no stress; NP + DS, no priming and drought stress; HP + DS, hydro priming and drought stress; AsA + DS, AsA priming and drought stress; Spd + DS, Spd priming and drought stress.

When compared with NP + NS, NP + DS significantly declined the IAA content of roots by 58.0% (**Figure 10A**). With exposure to drought stress, AsA priming and Spd priming treatment exhibited the highest IAA content, which was higher than no priming treatment by 113.5% and 132.8%. The drought stress down-regulated the relative expression of IAA biosynthetic genes like *OsYUC7*, *OsYUC11*, and *OsCOW1*, for no primed seedlings as compared with no water stress (**Figures 10B–D**). For priming treatment under water deficit, the expression levels of these genes were effectively up-regulated in both AsA and Spd priming, which were statistically comparable except for *OsYUC11* (**Figure 10C**). Compared with NP + DS, AsA priming maximized the expression of *OsYUC7*, *OsYUC11*, and *OsCOW1* by 56.5%, 36.4%, and 43.6%, respectively, whereas the respective increases in Spd priming were 57.7%, 47.8%, and 48.0%, respectively. Generally, seed priming with Spd is slightly higher than seed priming with AsA in terms of endogenous IAA concentration and relative mRNA level of investigated genes.

## 4 Discussion

### 4.1 Drought severely suppressed root development via different mechanisms

Drought, the primary abiotic stress that adversely influences root system architecture, has been investigated in depth in massive studies (Ye et al., 2018; Bi et al., 2021; Zhang et al., 2022b). In the current work, drought stress was detected to seriously suppress seedling growth, which was reflected by reduced elongation of seedlings and less biomass accumulation (**Figures 1**, **2**). Also, the root morphological parameters, i.e., total root length, specific root length, root surface, and root volume, were negatively modified under water-stressed conditions (**Figures 3**, **4**). The root tip is the main part of the plant to absorb water through the root system, and the more root tips, the greater the efficiency of moisture uptake. Studies have found that high IAA could enhance the growth and development of plant root hairs (Mangano et al., 2018). The present results observed a reduction in the number of root tips after drought treatment (**Figure 4D**), which may be due to the reduction of endogenous IAA content in the root system (**Figure 10A**). However, drought stress did not alter the average diameter of roots compared to normal conditions (**Figure 4C**), which is attributed to the greater drought tolerance of the WDR variety (Bi et al., 2021). Poor biomass and phenotypes of roots exposed to water deficit might be due to the reduction in water absorption (Karlova et al., 2021), blockage of mobility, and diffusion (He and Dijkstra, 2014).

Previously, many studies have investigated the macroscopic changes in root growth of rice seedlings under drought conditions. However, the mechanism of root drought resistance at the microscopic level during the seedling establishment has been rarely explored. It has been reported that transmission microscopy facilitated the detection of damage at the cellular level, offering the basis and insights into the potential impact of transmission electron macroscopic studies under non-biological stress (Shaw et al., 2014). In the current study, we found that root microstructure altered significantly because of drought stress, which was primarily exhibited in the deformation and shrinking of cells (**Figure 5B**), which was not conducive to the radial transport in the root system. Abnormal anatomical is commonly accompanied by alterations in cellular ultrastructure under stressful environments (Kamogashira et al., 2017). Consistent with the change in the anatomical structure of the root tips, water deficit seriously damaged the cell ultrastructure, mainly reflected in the unclear nucleolus, messy cytoplasm, and disappearance of different organelles (**Figures 6B, G**). Our present findings consistently matched the results of Fan et al. (2022), who reported that drought stress destroyed the structure of root tip cells in soybeans (Zhou et al., 2021). Furthermore, compared to normal conditions, drought stress reduced the number of mitochondria, which plays an important role in crop drought resistance during the early growth stage (Ma et al., 2017).

### 4.2 Seed priming is a low-cost technique for improving seedling growth and drought resistance

To overcome the growing challenges of water scarcity, researchers have employed numerous agro-management practices, for example, seed priming, foliar spray, and soil application. Foliar feeding involves the uptake of biostimulants through direct stomatal penetration and cuticular pores. This process is commonly effective during the day when the plant receives the most sunlight and the stomata and cuticle pores remain open for the maximum absorption of nutrients (Ronga et al., 2019). However, low surface cover of the crop during early growth led to a large proportion of applied foliar solutions falling straight onto the soil surface, resulting in wasted chemicals and reduced efficiency (Noack et al., 2010). Also, the quantity required is usually large, and it must be sprayed at regular intervals of growth phases, and phytotoxicity is a serious worry. Foliar spraying relies on several environmental factors, including temperature, humidity, and intensity of light. Soil application is a conventional method to produce a significant positive impact on agricultural productivity, yet the high application rates of agricultural products and the high cost required in the soil make this approach uneconomical in some situations. Furthermore, the majority of agricultural materials are chemical products, and their large-scale inputs raise diverse environmental concerns (Withers et al., 2020).

With the rapid global development of agriculture, direct seeding has emerged as an alternative to transplanting, offering labor and water savings of 20-30%. Seed priming facilitates synchronized seed growth in direct seeding. When compared to foliar spray and soil application, seed priming is more environmentally friendly and cost-effective, as it requires no chemical additives to the soil or air and is performed under controlled conditions before sowing. It also consumes fewer resources, whereas foliar spraying and soil treatment require large amounts of solution (Paul et al. 2017). Seed priming is a promising alternative method due to its low chemical usage, simple operation, low investment cost, high cost-effectiveness, and long-lasting effects (Veena and Puthur 2021). Seed priming directly acts on seeds by soaking them in appropriate solutions, which contributes to improving seed vitality, seedling emergence, and seedling growth, and effectively promoting plant resistance to abiotic stress and crop yield. For instance, the two-year study of Zulfiqar et al. (2021) found that seed priming further increased grain yield compared to foliar application or basal application (Zulfiqar et al. 2021). According to Zhang et al. (2022a), this technology improved rice yield by 19.0% in the Hubei province of China, where drought is a limiting factor for high-yield rice production. Our results also proved that seed priming promoted early seedling growth and tolerance to drought (**Figures 1** and **2**). A study on rapeseed cultivation in arid regions further confirmed these findings, showing that seed priming improved agronomic traits during the flowering stage under water-deficient conditions, and increased both grain yield and drought resistance in potted and field conditions (Khan et al. 2020).

Additionally, seed priming helps seedlings cope with environmental stress during early establishment and enhances the overall stress resistance of the plant (Yacoubi et al. 2011; Srivastava et al. 2021). Moderate abiotic stress can occur during dehydration and soaking, possibly due to inhibited radicle protrusion (Ashraf & Foolad, 2005). Plants can retain a memory of this stress, which may persist for weeks to months (Srivastava et al. 2021). Notably, seed priming only needs to be conducted once. During soaking, seeds absorb water and bind to protective and bioactive compounds, which positively affect seedling development, plant vigor, and survival under abiotic stress (Wiszniewska, 2021). Recent studies have shown that seed priming technology can enhance drought resistance in crops such as rice (Zhang et al. 2024), rapeseed (Khan et al. 2019), and wheat (Abid et al. 2018).

### 4.3 Comparison of the differences in the priming effects of AsA and Spd

The results revealed that AsA + DS and Spd + DS were more effective under water stress than other treatments. However, there are some variations in the priming effects of these two treatments. Seed priming with AsA showed drastic impacts on FW and DW in the shoot under stressful conditions (**Figures 2A, C**). Previous studies reported that AsA priming under drought conditions promoted seedling elongation and dry matter accumulation in various plants, such as wheat (Singh and Bhardwaj, 2016), alfalfa (Salemi et al., 2019), and pepper (Khazaei et al., 2020). On the other hand, the plants raised from seed primed with Spd had higher concentrations of total sugar and total protein (**Figures 7B, C**), lower degree of oxidative damage (**Figure 8**), and higher activity of POD and CAT (**Figures 9B, C**) when subjected to drought stress. Increasing evidence has proved that Spd functions as a signaling regulator in stress signaling pathways (Kasukabe et al., 2004), suggesting that it can directly take part in ROS scavenging and antioxidant defensive system. Previously, Li et al. (2014) found that Spd protects the plant from drought stress by modifying the antioxidant enzyme systems to prevent oxidative bursts (Li et al., 2014).

IAA, the major form of auxin in plants, functions in a wide range of cellular and developmental processes during plant lifespan, from promoting cell elongation, inducing division activity of cumbia cell, initiating architecture of root and leaf to contributing to flower and fruit development (Cao et al., 2019). Apart from modulating root system architecture, IAA is also involved in responses to diverse abiotic stresses, for example, salt stress (Zhang et al., 2021), high temperature (Blakeslee et al., 2019), and water deficit (Jung et al., 2015). Here, we found that pre-soaking with AsA and Spd led to a dramatic increase in endogenous IAA concentration in rice roots (**Figure 10A**). Within a certain range, improving IAA content is more beneficial for enhancing cell elongation, thereby stimulating root architectural traits and promoting root growth (Zhou et al., 2021). Likewise, one of the mechanisms by which IAA assists plants in resisting stress is the generation of new roots (Sreevidya et al., 2010).

The YUC pathway has been proposed as the most critical and major pathway to biosynthesize auxin in plant cells (Mashiguchi et al., 2011; Yoshikawa et al., 2014). *OsYUC7*, *OsYUC11*, and *OsCOW1*, belonging to the YUC gene family in rice, are key genes that participated in IAA synthesis, whose transcript levels are closely associated with IAA accumulation in roots (Woo et al., 2007; Qin et al., 2017; Zhang et al., 2018; Xu et al., 2021). Previous reports that auxin produced by the YUC pathway plays a crucial role in the root system formation of rice (Zhang et al., 2018). Also, studies have shown that the *OsYUC11* gene for auxin synthesis is upregulated in rice spikelets under drought stress (Sharma et al., 2018). Currently, the expression levels of the *OsYUC7* gene are relatively higher than that of other YUC family members (**Figure 10B**), which implies that *OsYUC7* may play a favorable regulating role in response to drought stress tolerance in plant roots. On the other hand, *OsYUC7*, *OsYUC11*, and *OsCOW1* were down-regulated in seedling roots under drought stress (**Figures 10B–D**), which could be the primary reason for the decrease in IAA under water-stressed conditions (**Figure 10A**). However, AsA priming and Spd priming upregulated these three genes under water deficiency (**Figures 10B–D**), and AsA + DS significantly upregulated *OsYUC11* than Spd + DS (**Figure 10C**). Both AsA and Spd could improve plant drought resistance by regulating the YUC gene, and AsA may have a greater regulatory effect.

## 5 Conclusion

**Figure 11:**
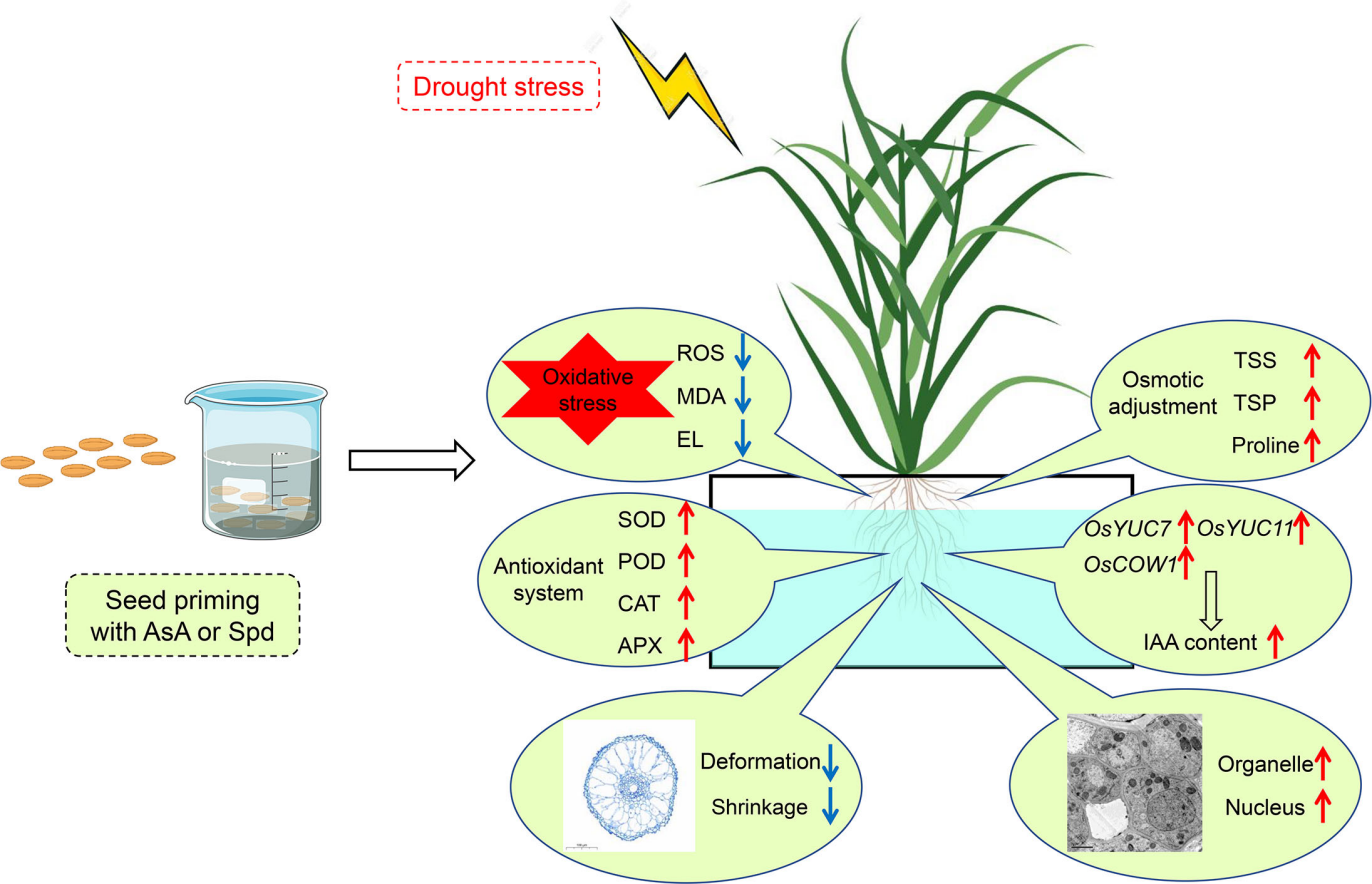
A model for the function of AsA or Spd priming agent under the drought stress response in rice plants. Red arrows represent increase, and blue arrows represent decrease.

In summary, drought stress resulted in ROS overproduction to induce oxidative stress, which ultimately suppressed the root growth of rice seedlings. Nevertheless, seed priming with AsA or Spd equally and effectively ameliorated root performance to resist water stress, including root elongation, below-ground biomass and root morphology. The ameliorative effects of these two priming treatments could be primarily correlated with (1) efficient ROS scavenging, (2) enhanced antioxidants activities, (3) accumulation of osmotic adjustment substances, (4) increased auxin contents and upregulation of IAA synthesis genes expression in rice roots (**Figure 11**). Likewise, some differences existed in the priming effects of these two priming agents. AsA priming drastically elevated FW and DW in the shoot, whereas Spd priming demonstrated higher contents of total sugar and total protein, reduced extent of oxidative injury, and higher activity of POD and CAT under stressful conditions. Combined with the alleviating impact of priming agents on the microstructure and ultrastructure of root cells, seed priming with AsA or Spd is of great value in imparting drought stress tolerance on rice roots during the seedling stage.

## Data Availability

The raw data supporting the conclusions of this article will be made available by the authors, without undue reservation.
