# Supplementary material for: Seed priming with ascorbic acid and spermidine regulated auxin biosynthesis to promote root growth of rice under drought stress
Source: Front Plant Sci. 2024 Dec 6;15:1482930. doi: 10.3389/fpls.2024.1482930 (PMC11658984; doi:10.3389/fpls.2024.1482930)
Supplement: Supplementary file 1 [file Table1.docx]

**Supplementary material:**

Additional supporting information may be found online in the Supplementary material section at the end of the article: Supplementary Table S1.

| **Supplementary Table S1 Sequences of primers used for qRT-PCR** | | | |
| --- | --- | --- | --- |
| Gene |  | Accession number | Sequence of the primers (5'-3') |
| *OsYUC7* | Forward | LOC_Os04g03980 | CAACGGATGGAAGGGTGAGT |
|  | Reverse |  | GCGGCTTAGAAGATGACGGA |
| *OsYUC11* | Forward | LOC_Os12g08780 | TGTGATGACAAAGGAGCTGATTC |
|  | Reverse |  | CCAACATCTATTACTGCTGACCG |
| *OsCOW1* | Forward | LOC_Os03g06654 | AAATCGACGGGATCTCCATGTT |
|  | Reverse |  | CTGCCCTGTTTGTAGGTTGTGC |
| *OsActin* | Forward | LOC_Os10g36650 | GCCATAAGTGCTACAGTAACCCA |
|  | Reverse |  | TTTAACCGGATGTCGGAAGG |
